# Genome sequence and transcriptomic profiles of a marine bacterium, *Pseudoalteromonas agarivorans* Hao 2018

**DOI:** 10.1038/s41597-019-0012-y

**Published:** 2019-03-27

**Authors:** Kai Shan, Chunlei Wang, Wenlin Liu, Kai Liu, Baolei Jia, Lujiang Hao

**Affiliations:** 1 State Key Laboratory of Biobased Material and Green Papermaking, Qilu University of Technology (Shandong Academy of Sciences), Jinan, 250353 China; 2 School of Bioengineering, Qilu University of Technology (Shandong Academy of Sciences), Jinan, 250353 China

**Keywords:** Genome, Marine microbiology, DNA sequencing

## Abstract

Members of the marine genus *Pseudoalteromonas* have attracted great interest because of their ability to produce a large number of biologically active substances. Here, we report the complete genome sequence of *Pseudoalteromonas agarivorans* Hao 2018, a strain isolated from an abalone breeding environment, using second-generation Illumina and third-generation PacBio sequencing technologies. Illumina sequencing offers high quality and short reads, while PacBio technology generates long reads. The scaffolds of the two platforms were assembled to yield a complete genome sequence that included two circular chromosomes and one circular plasmid. Transcriptomic data for *Pseudoalteromonas* were not available. We therefore collected comprehensive RNA-seq data using Illumina sequencing technology from a fermentation culture of *P. agarivorans* Hao 2018. Researchers studying the evolution, environmental adaptations and biotechnological applications of *Pseudoalteromonas* may benefit from our genomic and transcriptomic data to analyze the function and expression of genes of interest.

## Abstract (summary)

**Table:** 

Design Type(s)	transcription profiling design • source-based data analysis objective
Measurement Type(s)	whole genome sequencing assay • transcription profiling assay
Technology Type(s)	DNA sequencing • RNA sequencing
Factor Type(s)	
Sample Characteristic(s)	Pseudoalteromonas agarivorans • ocean biome

Machine-accessible metadata file describing the reported data (ISA-Tab format)

## Background & Summary

Bacteria in the marine environment are an essential component of marine ecosystems^1^. Those bacteria inhabit diverse environments, including seawater, deep-sea and hydrothermal vents, and polar environments. Marine bacteria can colonize external envelopes and/or enter internal tissues of marine plants and animals; certain bacteria can establish highly symbiotic relationships with their specific host organisms^2^. Considering that marine bacteria inhabit a wide range of niches with pervasive and dynamic chemical and physical gradients, they use various mechanisms for physiological adaptations, such as osmolyte accumulation, unique structures of biomacromolecules, and production of natural bioactive metabolites and exopolysaccharides^3^. These distinctive properties of marine bacteria have attracted interest since they have potential for use in industry, including in pharmaceuticals, cosmetics, agrichemicals, nutritional supplements, molecular probes, metabolites/compounds, enzymes and fine chemicals^4–6^.

The members of the genus *Pseudoalteromonas*, which is gram-negative, aerobic, non-fermentative, heterotrophic, and halophilic, can be considered to be obligate marine microorganisms. This genus now contains more than 40 validly described species^7^. These species are widespread in marine microbial ecosystems and produce a large number of biologically active substances^8^. *Pseudoalteromonas* generally display agarolytic, bacteriolytic, antibacterial, and algicidal activities. Interestingly, the strains of this genus are routinely found to be associated with marine eukaryotes, such as tunicates^9^, algae^10^, sponges^11^, mussels^12^, pufferfish^13^, and marine plants^14,15^. *Pseudoalteromonas* produce a series of chemicals that are active against the target organisms, which may provide benefits for the bacteria regarding nutrient competition and colonization^8^. *Pseudoalteromonas* also can produce diverse extracellular enzymes that are of potential biotechnological interest, such as protease^16^, phospholipase^17^, agarose^18^, and collagenase^19^. Considering the potential applications and frequent occurrence of members of this genus, *Pseudoalteromonas* has attracted great interest from many researchers.

Due to advances in high-throughput DNA sequencing platforms, including Roche 454, Illumina, SOLiD, Ion Torrent, and PacBio^20^, researchers can better understand the function, evolution, environmental adaptation and potential application of marine bacteria. Complete genome sequences of several strains of *Pseudoalteromonas* have been completed^21^. In the species of *Pseudoalteromonas agarivorans*, only 4 strains have been sequenced and the data have been deposited in Genbank database by the end of 2018, which are *P. agarivorans* S816, *P. agarivorans* NW 4327, *P. agarivorans* DSM 14585, and the strain in the current study (*P. agarivorans* Hao 2018). Among them, only the genome sequences of *P. agarivorans* DSM 14585 and *P. agarivorans* Hao 2018 have been assembled completely. In this research, the genome of *P. agarivorans* Hao 2018 was sequenced using Illumina and PacBio technologies. The advantages of PacBio sequencing are the resulting both long read length (>10 Kb) and highly contiguous *de novo* assembly, which can close gaps in the assembled genome sequence and allow readthrough of repetitive regions. However, the gene expression profile of *Pseudoalteromonas* has not been studied. Transcriptomic analysis using Illumina HiSeq was performed to gain a comprehensive understanding of gene expression of *P. agarivorans* Hao 2018. The dataset reported in this study will be useful for analysing the evolution and environmental adaptation of *Pseudoalteromonas* and for discovering newly applicable genes and biomolecules.

## Methods

### Genomic DNA preparation

*P. agarivorans* Hao 2018 was isolated from an abalone breeding environment in Rongcheng, Shandong Province, China. The strain was cultivated in Zobell 2216E solid medium containing 5.0 g/L peptone, 1.0 g/L yeast extract, 0.01 g/L sodium phosphate, and 35 g/L bay salt, pH 7.6~7.8, at 25 °C. Genomic DNA of the strain was extracted using a Microbial DNA extraction kit (Takara, Tokyo, Japan) according to the manufacturer’s instructions. The DNA quality was evaluated using 1% agarose gel electrophoresis and a Nanodrop spectrophotometer (Thermo Fisher Scientific, Waltham, MA). The purified genomic DNA should be visible as a single band of approximately 50 Kb in agarose gel. By nanodrop spectrophotometer, the optical density ratio 260/280 nm and 260/230 nm should be >1.8 and >2.0, respectively.

### Genome Sequencing

Two different gDNA libraries were constructed according to the manufacturers’ instructions of the Illumina Miseq system and PacBio system. Genomic DNA sequencing was performed on Illumina Miseq and PacBio RS II platforms by Personalbio Biotechnology (Shanghai, China). For Illumina Miseq sequencing, genome DNA were first fragmented by the Covaris M220 sonicator (Covaris, Woburn, USA). Illumina TruSeq DNA Sample Prep Kit (Illumina, USA) was then employed for the generation of short-insert (400 bp) libraries with the steps of bead-based size selection, end-reparation, adenylation, ligation into Illumina sequencing adapters, and enrichment as following manufacturer’s instructions. The libraries were evaluated using Pico-Green (Quant-iT, Invitrogen, USA) and Bioanalyzer DNA 1000 kit (Agilent Technologies, USA). The libraries were sequenced by the Illumina Miseq system with V3 chemistry. For PacBio RS II sequencing, DNA samples was fragmented using Covaris g-TUBE shearing device and then purified with AMPure beads (Beckman Coulter, USA). The fragmented genomic DNA was used to construct the library with the PacBio SMRTbell library preparation kit. Briefly, the fragmented DNA was firstly repaired via ExoVII. After cleaning by AMPure beads, the blunt-end of DNAs were ligated with the blunt hairpin adapters and the DNA without adaptors were digested by ExoIII and ExoVII enzymes. Finally, the 20 kb SMRTbell libraries was selected using BluePippin and were sequenced using the PacBio RS II system with V2 chemistry. The read qualities were examined by FastQC (http://www.bioinformatics.babraham.ac.uk/projects/fastqc/).

### Genome assembly

Raw reads from the Illumina Miseq system were quality filtered and error corrected with SOAPEC (kmer = 17)^22^. The reads from the PacBio system were assembled by the hierarchical genome-assembly process 4 (HGAP4) pipeline (Pacific Biosciences, SMRT Link 5.0)^23^. In the pre-assembly step, the raw reads were firstly filtered by the pipeline using default settings with read quality (rq) of ≥0.7. Then, *de novo* assembly was performed using the FALCON in the HGAP4 tools with Seed Coverage = 60. In the assembly polishing step, the Best Algorithm was used to polish the genome assembly. The filtered Illumina short reads were aligned to the *de novo* assembled contigs with the Burrows-Wheeler Alignment tool^24^. The alignment results were sorted by Picard (http://broadinstitute.github.io/picard/), followed by base quality recalibration with Pilon^25^. The final contigs were subsequently circularized by Circlator version 1.5.5^26^.

### Genome annotation

The complete genome sequence of *P. agarivorans* Hao 2018 was submitted to the NCBI Prokaryotic Genomes Automatic Annotation Pipeline (PGAAP) for auto-annotation. The tRNAscan-SE^27^ and RNAmmer^28^ software programs were used to annotate tRNA and rRNA genes, respectively. Genes were also annotated by aligning with sequences in other publicly available protein databases, including the NCBI non-redundant protein (Nr) database, protein families (Pfam), UniProt/Swiss-Prot database, gene ontology (GO), cluster of orthologous groups of proteins (COG), and KEGG ontology^29^.

### RNA extraction and sequencing

Fermentation of *P. agarivorans* Hao 2018 was carried out with triple repeats at 25 °C in a medium containing 45 h/L glucose, 2.5 g/L (NH_4_)_2_SO_4_, and 45 g/L bay salt, pH 7.6~7.8. Cells were cultured for 2 and 24 h, and the samples were identified as F2-(1-3) and F24-(1-3), respectively. Fermented cells were harvested by centrifugation at 10,000 × *g* for 5 min at 4 °C. Total RNA from the cells was extracted using TransZol reagent (TransGen Biotech, Beijing, China) according to the manufacturer’s instructions. RNA quality was evaluated using an Agilent Bioanalyzer 2100 system (Agilent Technologies, USA). Paired-end libraries were prepared for each RNA-seq sample using a Truseq Stranded Total RNA Sample Preparation Kit (Illumina). After quality control, all of the libraries were sequenced using an Illumina HiSeq 2500 instrument (Personal Biotechnology, Shanghai).

### Analyses of RNA-Seq data

The read qualities were checked by FastQC. FASTX-Toolkit program was used to filter out rRNA reads, sequencing adapters, short-fragment reads and other low-quality reads from raw reads of the sequences (http://hannonlab.cshl.edu/fastx_toolkit/). The remaining clean reads were mapped to *P. agarivorans* Hao 2018 genome based on a local alignment algorithm using Bowtie2 software^30^. The expression levels were normalized by calculating the fragments per kilobase million reads (FPKM) value^31^. The differential expression of the transcripts was calculated using DESeq software^32^.

## Data Records

### Genome

All of the raw reads from Illumina and PacBio sequencing were deposited in the NCBI Sequence Read Archive^33^. The complete genome sequences and the annotated genes for the chromosome_1, chromosome_2, and plasmid of *P. agarivorans* Hao 2018 were deposited in GenBank^34^.

### Transcriptome

The transcriptome sequencing data were deposited in the NCBI Sequence Read Archive^35^.

## Technical Validation

### Genomic data validation

**Fig. 1:**
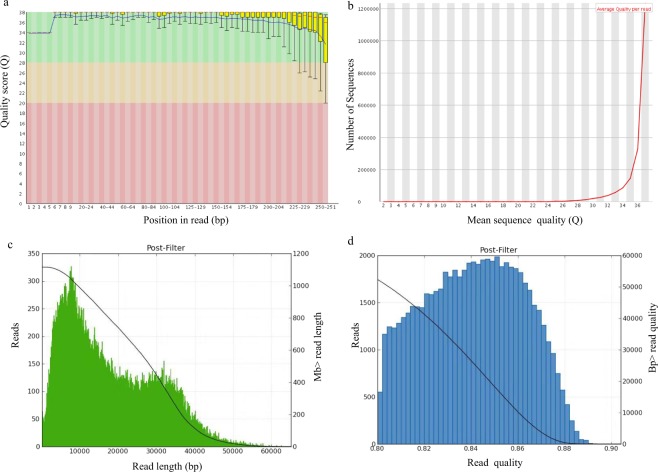
Quality control of the genome sequencing data. (**a**) Base quality using Phred scores for the data from Illumina Miseq sequencing. In the box plots, the whiskers represent the range between the 10 and 90% quantiles; the yellow boxes show the range between the upper and lower quartiles; the red lines represent median. The blue line indicates the mean quality. (**b**) Distribution of read-over-read quality. (**c**) Read length distribution of PacBio sequencing after filtering. (**d**) Read quality distribution of PacBio sequencing after filtering.

Members of the family Pseudoalteromonadaceae play important ecological roles in marine environments and are highly adaptable to a variety of ecological habitats. *P. agarivorans* Hao 2018 has also been isolated from marine environments. This strain was sequenced by using both Illumina and PacBio^33^ technologies to explore its biotechnological potential and mechanisms of adaptation to environments. The quality of the sequencing reads from the Illumina platform was assessed with FastQC. The quality scores of the reads were quite stable across the reads and 92% of the called bases had a Phred score ≥30, indicating the base quality is high enough. Even the ends of the reads, which are known to be of lesser quality, were still within the Q20 range (Fig. 1a). For an additional quality evaluation of the sequencing run for the samples, histograms of the numbers of reads versus the average read quality were generated (Fig. 1b) and showed that the quality of most of the reads was around Q37. PacBio sequencing generated 62,879 sequence reads having an average read length of 17,787 bp (Fig. 1c) and achieving an accuracy of 0.839 after removing the adapter (Fig. 1d). These results suggested that the sequencing data were good enough for downstream analyses.

**Fig. 2:**
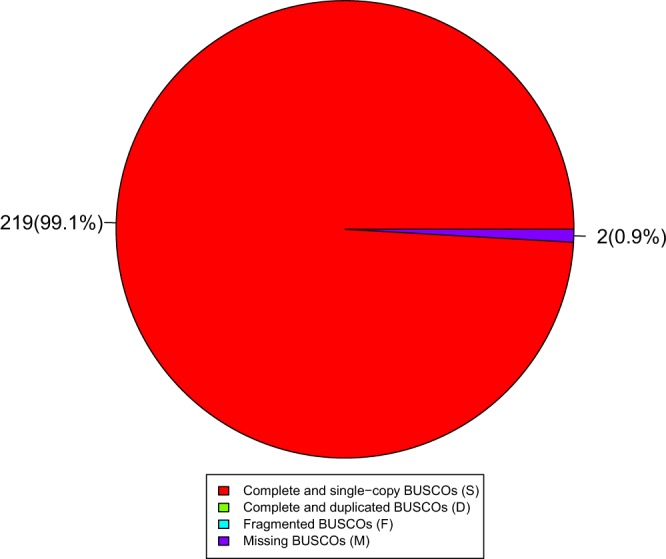
Assessment of the completeness of the *Pseudoalteromonas agarivorans* Hao 2018 genome by BUSCO.

Replicates, duplicates, and sequences of low quality or sequences with short read lengths (<100 bp) from the Illumina platform were filtered out of the raw data. The filtered reads were used to improve the *de novo* assembly from Pacbio reads. The improved assembly was circularized and verified using Circlator 1.5.5^26^. The final assembly was 99.1% complete with 0.9% of the sequence predicted to be missing as estimated by BUSCO^36^ (Fig. 2).

**Fig. 3:**
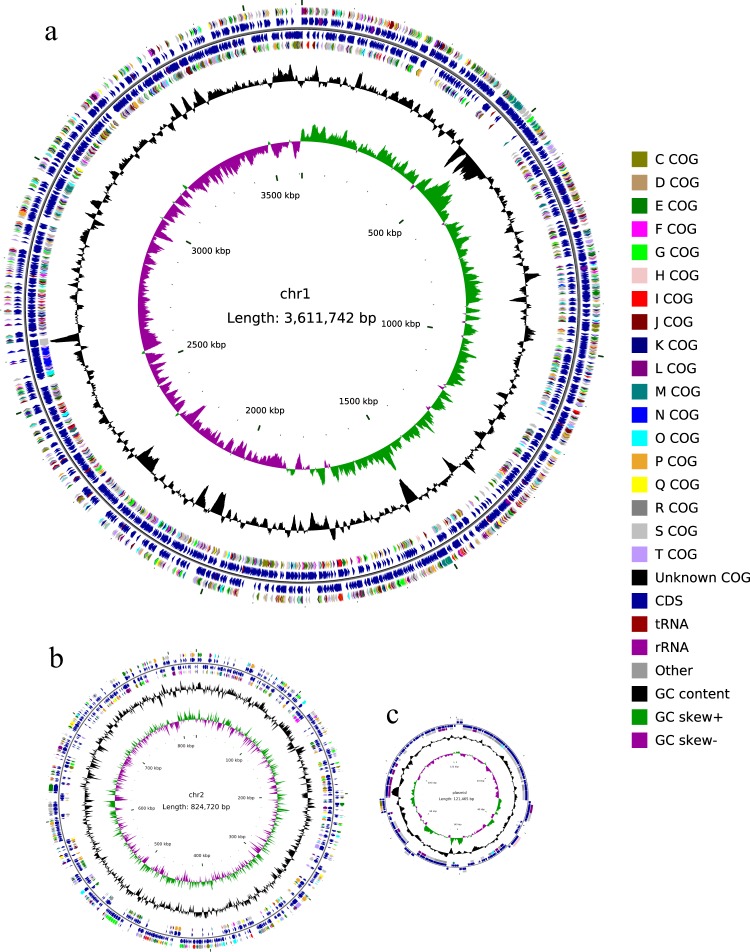
Circular representation of the genome of *Pseudoalteromonas agarivorans* Hao 2018. (**a**) Chromosome_1; (**b**) chromosome_2; (**c**) plasmid. The outer two rings (ring 1 and ring 2) represent the annotated genes encoding proteins and structural RNAs on the plus and minus strands, respectively. The colours in the two rings represent the COG categories of the genes. The ring 3 (black circle) indicates the GC content (%), and the ring 4 depicts the GC skew.

The genome of the strain is composed of two circular chromosomal genomes (chr_1 and chr_2) and one circular plasmid (^34^, Fig. 3). Chr_1 (3,611,742 bp), which has an average GC content of 41.03%, contains 3,234 predicted open reading fragments (ORFs), 26 rRNA genes, and 102 tRNA genes encoding 20 amino acids. Chr_2 (824,720 bp), which has an average GC content of 40.30%, contains 747 predicted ORFs without any rRNA genes or tRNA genes. The plasmid (121,465 bp), which has an average GC content of 39.30%, contains 126 predicted ORFs.

### Transcriptomic data validation

**Fig. 4:**
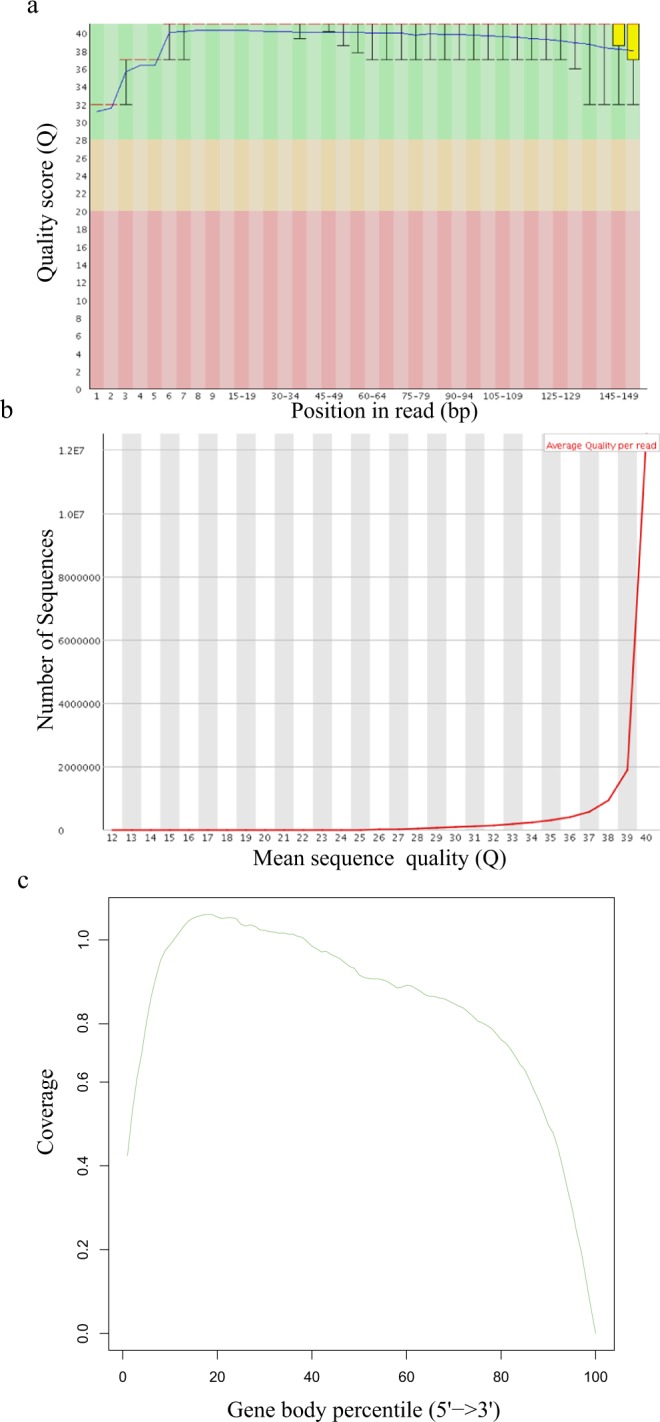
Quality of the transcriptomic reads derived from the Illumina sequencing data. (**a**) Base quality using Phred scores for the transcriptomic data using F2-1 as a representative sample. In the box plots, the whiskers represent the range between the 10 and 90% quantiles; the yellow boxes show the range between the upper and lower quartiles; the red lines represent median. The blue line indicates the mean quality. (**b**) Distribution of read-over-read quality using F2-1 as a representative sample. (**c**) Gene body coverage by plotting read depth from 5’ to 3’ across the genes using the image of sample F2-1 as a representative of all samples.

The genus *Pseudoalteromonas* is extensively studied due to the production of biomolecules with industrial interest. The gene expression profiles of *P. agarivorans* Hao 2018 were studied after fermentation for 2 h and 24 h with triple repeats. The quality of purified RNA was verified using an Agilent Bioanalyzer 2100 system (Agilent Technologies) before construction of cDNA library and sequencing. Six cDNA libraries were obtained during fermentation and were prepared and sequenced using the Illumina HiSeq platform^35^. Finally, 204,091,156 reads with an average sequence length of 150 bp were obtained (Table 1). The quality of the 6 samples was also assessed using the same methods. The transcriptomic data of F2-1 were used as a representative example to show the quality of the data (Fig. 4a,b). The reads obtained were of very high quality, as more than 97% of the reads across the conditions reached a Phred score greater than Q20, while more than 94% of reads were above the Q30 threshold. In addition, the reads evenly spread along the length of the gene body as shown by the gene coverage graph of sample F2-1(Fig. 4c). Only the analysis of sample F2-1 is shown because the gene coverage of the other samples is highly comparable to that of F2-1. The reads were further cleaned with FASTX-Toolkit to remove sequencing adapters and the low-quality reads. The percentage of clean reads was greater than 99% (Table 1). These data indicated that the high-quality transcriptomic reads were suitable for downstream analyses.

**Table 1:** Summary of the Illumina sequencing-derived transcriptomic data sets of the *Pseudoalteromonas agarivorans* Hao 2018 fermentation samples at 2 h (F2) and 24 h (F24).

Sample	Reads (No.)	Bases (bp)	Q20 (%)	Q30 (%)	Clean Reads (No)	Clean Data (bp)	Clean Reads (%)
F2-1	35,628,738	5,344,310,700	98.05	94.97	35,539,354	5,315,365,568	99.74
F2-2	32,918,020	4,937,703,000	97.95	94.8	32,827,394	4,911,816,504	99.72
F2-3	30,234,438	4,535,165,700	97.95	94.77	30,147,348	4,510,663,522	99.71
F24-1	36,639,772	5,495,965,800	97.71	94.31	36,407,882	5,448,102,540	99.36
F24-2	32,329,744	4,849,461,600	98.4	95.7	32,234,184	4,824,059,398	99.70
F24-3	36,340,444	5,451,066,600	98.35	95.62	36,228,242	5,419,476,838	99.69

## ISA-Tab metadata file

Download metadata file
